# Analysis of the Effect of Various Potential Antimicrobial Agents on the Quality of the Unpasteurized Carrot Juice

**DOI:** 10.3390/molecules28176297

**Published:** 2023-08-28

**Authors:** Katarzyna Ratajczak, Agnieszka Piotrowska-Cyplik, Paweł Cyplik

**Affiliations:** 1 Department of Food Technology of Plant Origin, Poznan University of Life Sciences, Wojska Polskiego 31, 60-624 Poznań, Poland; agnieszka.piotrowska-cyplik@up.poznan.pl; 2 Department of Biotechnology and Food Microbiology, Poznan University of Life Sciences, Wojska Polskiego 48, 60-627 Poznań, Poland; pawel.cyplik@up.poznan.pl

**Keywords:** food microbiology, food safety, microbial quality of carrot juice, carrot juice microbiome

## Abstract

Short shelf-life and poor microbial quality of minimally processed foods of plant origin pose a serious problem for the food industry. Novel techniques of minimal treatment combined with disinfection are being researched, and, for fresh juice, the addition of antimicrobial agents appears to be a promising route. In this research, fresh, nonfiltered, unpasteurized carrot juice was mixed with four potential antimicrobials (bourbon vanilla extract, peppermint extract, cannabidiol oil, and grapefruit extract). All four variants and the reference pure carrot juice were analyzed for metapopulational changes, microbial changes, and physicochemical changes. The potential antimicrobials used in the research have improved the overall microbial quality of carrot juice across 4 days of storage. However, it is important to notice that each of the four agents had a different spectrum of effectiveness towards the groups identified in the microflora of carrot juice. Additionally, the antimicrobials have increased the diversity of the carrot juice microbiome but did not prevent the occurrence of pathogenic bacteria. In conclusion, the use of antimicrobial agents such as essential oils or their derivatives may be a promising way of improving the microbial quality and prolonging the shelf-life of minimally processed foods, such as fresh juices, but the technique requires further research.

## 1. Introduction

Consumption of fresh fruits and vegetables is a crucial part of the human diet, as they are a source of a wide variety of nutrients and bioactive compounds. Carrot (*Daucus carota* L.) is one of the most popular vegetables in the food industry. The main nutritional components in carrots are sugars, primarily simple sugars (such as glucose, fructose, and sucrose). In addition, the high content of carotene means that carrots are one of the main sources of provitamin A in the human diet. Carotenoids, including α- and β-carotene, have antioxidant properties and show anti-cancer abilities [1,2,3]. The majority of the world-wide yearly yield (around 82%) of carrot is consumed raw or minimally processed. Minimally processed carrot is usually found in the form of slices, cubes, or shredded carrot. However, one of the more popular forms is carrot juice, especially unpasteurized juice. Fresh carrot juice is among the most popular vegetable beverages in the world [4]. Carrot juice retains the majority of fresh carrot’s bioactive compounds, such as carotenoids, vitamins, and minerals. It is also low in sugar and high in fiber content [4,5]. Commercial carrot juice available in the stores has often been thermally processed (pasteurized); sometimes, it also contains acidifying additives. Such processing techniques negatively affect the final product, as they change the texture and taste of the juice, and are detrimental to the juice’s pro-health properties. However, unpasteurized carrot juice is susceptible to microbiological degradation due to its liquid form and low acidity, making access to nutrients very easy for the microflora naturally occurring on carrots. Usually, the initial microbial contamination of fresh vegetable or fruit juices already ranges from 3 to 5 log CFU/mL. The spoilage of juice is caused most often by yeasts, molds, and lactic acid bacteria, but these are not the only microorganisms found in fresh juices. Groups of Enterobacteriaceae and Pseudomonadaceae are prominent in fresh carrots, and, therefore, also in fresh carrot juice. Several cases of foodborne diseases connected with fresh, untreated vegetable juices have been recorded in the last decade. Many pathogenic microorganisms, such as *Salmonella* sp., *Listeria monocytogenes*, strains of *Escherichia coli,* including O157:H7, *Bacillus cereus*, and others have been found in fresh produce, including carrots [6]. The interest in research on potential alternative, non-thermal methods of inactivating microorganisms and their enzymes while maintaining the original properties of fresh produce has increased. The ultimate goal is to obtain a high-quality product with all its bioactive compounds preserved that has a significantly prolonged shelf-life and will be both appealing and safe to the customer [4,6,7,8,9].

An interesting alternative method of minimal processing available in the production of unpasteurized carrot juices is natural compounds with antimicrobial properties. Natural antimicrobial agents are usually extracts, oils, or specific compounds obtained from plants. These antimicrobials are usually classified as GRAS (generally recognized as safe) in regard to their use in the food industry and are able to satisfy the increasing consumer demand for natural preservatives. Often, such agents, for example, essential oils (EOs) or extracts, already have a history of usage in the food industry, cosmetics, or medicine because of their flavor, scent, and potential health benefits [10,11]. Research has proved that the use of natural antimicrobial agents affects both Gram-positive and Gram-negative bacteria present in fresh juices. However, Gram-positive bacteria are usually more susceptible to disruption by essential oils. It has already been proven that the use of essential oils can reduce the heat level used in thermal treatment or eliminate the thermal treatment of vegetable or fruit juice. Essential oils have been proven to be effective against both planktonic bacteria and biofilms in fresh juices [12,13]. The mechanism of inhibition of microbial growth differs between various agents. Some of them rely on membrane sensitization to stress factors (e.g., to heat) and increasing the membrane permeability. However, antimicrobials such as essential oils most often disrupt the cell wall and membrane structure and cause the destabilization of the cytoplasmic environment of microorganisms, which results in reducing their metabolic activity, leading to apoptosis [10,12,13,14,15].

Essential oils are hydrophobic, volatile liquids obtained from various plants via distillation or extraction, which may present diverse bioactive properties depending on their composition [16]. They are often simultaneously used as flavoring agents, antimicrobial agents, and preservatives in the food industry; however, their use remains somewhat challenging due to the strong aroma and flavor, which may be unacceptable for the customer, and due to their poor water solubility. This raises the need for new solutions regarding the application of essential oils in food products. Essential oils consist of two fractions: volatile (up to 99%) and non-volatile, and, in each of them, different compounds (including hydrocarbons, fatty acids, terpenes, flavonoids, waxes, and many others) can be found. The antimicrobial properties of each essential oil depend on its composition, and while one main mechanism of action can be determined for each EO, in reality, the cell inactivation occurs through a series of reaction caused by the EO within it [12,13]. Among the studied possible antimicrobial agents applicable in the food industry is vanilla essential oil and vanilla extract. Vanillin (4-hydroxy-3-methoxybenzaldehyde) is the main phenolic compound found in seedpods of the vanilla plant (*Vanilla planifolia* A.). Vanillin has both antioxidant activity and antimicrobial activity and is especially effective as an inhibitor of yeasts and molds. The vanilla essential oil has been proven to exhibit antimicrobial activity against *Enterobacter aerogenes*, *Escherichia coli*, *Proteus vulgaris*, *Pseudomonas aeruginosa*, and *Streptococcus faecalis* during in vitro tests, and it also has inhibited the expression of quorum sensing genes of soil Gram-negative bacteria [17]. The ability of vanillin to cause thermosensitization of *E. coli* has also been researched and proven in carrot juice [11,18]. Another example of commonly used essential oil is various citrus EOs. Grapefruit (*Citrus paradisi* Macf.) essential oil presents antimicrobial properties, as well as antioxidant, diuretic, and antiseptic properties [19]. Grapefruit essential oil also inhibited yeast growth during the fermentation of wort [20]. During in vitro tests, grapefruit EO has shown antimicrobial activity against *Bacillus subtilis*, and also against *Escherichia coli*, *Salmonella typhimurium*, and *Staphylococcus aureus* [21]. Citrus essential oils (CEOs) are commonly used in the food industry and cosmetics because of their scent and taste. CEOs also exhibit broad antibacterial, antifungal, and insecticidal properties (e.g., against *Bacillus subtilis* and *Staphylococcus aureus*) through mechanisms of cell membrane disruption. Their composition constitutes mostly derivatives of terpenoids and non-terpenoids, including bioactive functional compounds, from which one of the most common is limonene, the predominant monoterpene in grapefruit essential oil [21,22]. Various spice essential oils present antimicrobial and antifungal activity against pathogens such as *Salmonella* sp., *Listeria monocytogenes*, *Bacillus cereus*, *Klebsiella pneumoniae*, and *Staphylococcus aureus* [12]. Another potential antimicrobial agent, mint essential oil, is obtained from the mint plant (*Mentha* sp. L.). Mint essential oil exhibits antimicrobial, antifungal, and antiviral properties against many microorganisms, including pathogens such as *Listeria monocytogenes* and *Staphylococcus aureus;* both peppermint oil and spearmint oil have exhibited great antimicrobial activity against *Bacillus subtilis*, *Vibrio* spp., and a wide variety of Gram-negative bacteria. The main components responsible for the bioactive properties of mint EO are L-menthol, menthone, menthyl acetate, and limonene, which disrupt the cell’s membrane by changing its pH gradient, leading to the cell’s death. Many plants from the mint family (this includes *M. canadensis*, as well as *M. piperita* and *M. spicata*, and *M. aquatic*), as well as their extracts and essential oils, have been commonly used in traditional herb treatments for centuries, and are still widely used in the pharmaceutical, food, and cosmetic industries [13,23,24,25]. It is important to note that most essential oils, and their compounds, are recognized as GRAS by USFDA and are allowed for use in the food industry by the European Commission [7]. However, due to their high reactivity, hydrophobic nature, and possible interactions with carbohydrates and fatty acids in food products, the use of EOs is sometimes considered impractical in pure form. Often, natural essential oils are used in the forms of microcapsules in food or incorporated into polymer matrices used in packaging; however, it might be possible to use them in diluted forms of extracts [26].

An interesting novel antimicrobial agent is cannabidiol (CBD) oil obtained from the hemp plant (*Cannabis sativa*). Cannabinoids, a group of substances extracted from the cannabis plant to which cannabidiol belongs, have a long history of medicinal and therapeutic use. While some cannabis compounds have an adverse effect on human health (including impaired motor skills, anxiety, addiction, and deficits in neurocognitive functioning), CBD itself is considered safe, exhibiting positive, non-psychoactive effects on human health. Cannabidiol, like most cannabinoids, exhibits antimicrobial properties, including against such pathogens as *Bacillus* spp., *Enterococcus* spp., and *Listeria monocytogenes* [27]. CBD oil has been noted as an effective antimicrobial agent against bacterial strains of *Neisseria meningitidis*, *Neisseria gonorrhoeae*, *Enterococcus faecium*, *Clostridioides difficile*, *Listeria monocytogenes*, *Staphylococcus aureus*, and *Salmonella typhimurium*, among others. It is important to note that most research aimed at CBD oil analysis is conducted in vitro and that the mechanism of cannabidiol’s antimicrobial action is not yet fully confirmed [28,29]. Clinical trials proved CBD to be effective against *Staphylococcus aureus* nasal infections. Cannabidiol is considered to be a member of a new class of antimicrobial compounds exhibiting activity against new pathogens, often ones with antibiotic resistance [30].

Based on the above, the main goal of this study was the analysis of the possibility of prolongation of shelf-life of fresh carrot juice through the addition of various natural antimicrobial agents. The goal was obtained through analysis of changes in the microbiome of unpasteurized carrot juice, with potential antimicrobial agents (bourbon vanilla extract, peppermint extract, CBD oil, and grapefruit extract) added, during storage at 6 °C.

## 2. Results

### 2.1. Microbiome Analyses

#### 2.1.1. Next-Generation Sequencing Analysis

**Figure 1:**
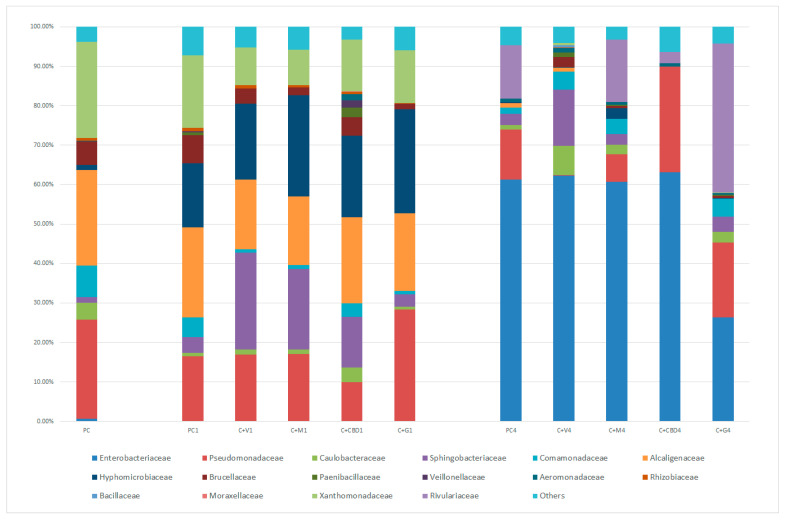
Relative abundance of bacteria at the family level across carrot juice variants during 4 days of storage.

**Figure 2:**
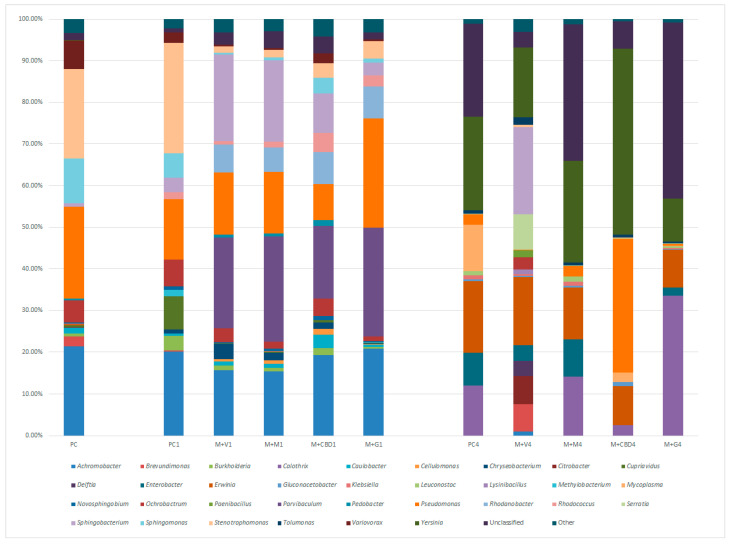
Relative abundance of bacteria at the genus level across carrot juice variants during 4 days of storage.

Next-generation sequencing of bacterial 16S rRNA genes isolated from carrot juice samples allowed for the detection of a total of 435 genera, 16 families, and 6 phyla. The relative abundance of major bacterial families and genera are shown in Figure 1 and Figure 2, respectively.

The dominant phylum across all carrot juice samples was Proteobacteria. In pure carrot juice on the day of preparation, over 95% of all identified bacteria belonged to Proteobacteria; however, both storage and addition of potential antimicrobials affected the microbiome composition. In pure carrot juice after 24 h of storage, Proteobacteria had a 90% share in the total microbiome of juice, and, after 4 days of storage, Proteobacteria had an 81% share in the total microbiome of juice. After 1 day of storage, a significant increase in the percentage of bacteria belonging to the Bacteroidetes and Actinobacteria phylum was observed across all carrot juice variants, including pure carrot juice. Interestingly, the number of bacteria belonging to these two phyla decreased significantly during the remaining time of storage. After 4 days of storage, bacteria from phyla Firmicutes and Cyanobacteria were detected in all carrot juice samples.

Figure 1 presents the relative abundance of bacterial families identified in carrot juice samples. In fresh, pure carrot juice, three predominant families can be observed: Pseudomonadaceae, Alcaligenaceae, and Xanthomonadaceae, whose combined share of abundance was over 70%. After 24 h of storage, the Hyphomicrobiaceae family became the fourth most dominant bacterial group. Interestingly, after 4 days of storage, the pure carrot juice microbial profile had changed significantly. The most prevalent family was Enterobacteriaceae at 61% of relative abundance; the second most abundant family was Rivulariaceae.

Moreover, the addition of potential antimicrobial agents affected the microbiome of carrot juice over the course of its storage. All antimicrobials caused an increase in the abundance of the Sphingobacteriaceae and Hyphomicrobiaceae families. Simultaneously, a decrease in the abundance of the Comamonadaceae, Brucellaceae, Alcaligenaceae, and Xanthomonadaceae families was noted. Variants with vanilla or mint extract added had a relatively similar microbial profile and caused the most notable changes in the abundance of the Xanthomonadaceae and Sphingobacteriaceae families. CBD oil and concentrated grapefruit extract had the greatest influence on the Pseudomonadaceae family. CBD oil appeared to have the strongest inhibitory effect on the Pseudomonadaceae family. However, after 4 days of storage, the C+G variant was the only one in which the Enterobacteriaceae family was not highly prevalent. In the C+V variant, the growth of the Rivulariaceae and Pseudomonadaceae families was noticeably inhibited, while, in the C+G variant, these families’ relative share in the microbiome increased.

Of 435 genera identified across all samples, 33 could be classified as most prevalent in the microbiome of carrot juice variants, and their abundance is presented in Figure 2. At the time of preparation, the most dominant genera in pure carrot juice were *Pseudomonas*, *Achromobacter*, and *Stenotrophomonas* (abundance over 20%). All three of these bacteria can be commonly found in soil and groundwater, and, among them, potentially pathogenic organisms can be found. After the first 24 h of storage, a decrease in the abundance of *Pseudomonas* spp. and an increase in the *Stenotrophomonas* spp. were observed. After 4 days of storage, *Achromobacter* bacteria were not identified in pure carrot juice; however, a significant increase in the *Calothrix*, *Enterobacter*, *Erwinia*, *Mycoplasma*, and *Yersinia* spp., as well as the rise in the unclassified bacteria abundance, was noted. Bacteria of the *Erwinia* genus are often plant pathogens; however, *Enterobacter*, *Mycoplasma*, and *Yersinia* are pathogenic or potentially pathogenic. *Mycoplasma*, in particular, could be noted for its intrinsic antibiotic resistance.

The addition of potential antimicrobials has caused an overall decrease in the abundance of *Stenotrophomonas* spp. and, in the case of vanilla and mint extract, also a significant decrease in the *Achromobacter* spp. abundance. With the exception of concentrated grapefruit extract, all antimicrobials also decreased the prevalence of *Pseudomonas* spp. Interestingly, all four antimicrobial agents caused an increase in the abundance of the *Parvibaculum* genus, which belongs to a larger order of soil bacteria commonly found in the plant rhizosphere. After 4 days of storage, *Achromobacter* spp. abundance had decreased significantly, similar to its behavior in pure carrot juice. However, while both *Enterobacter* and *Erwinia* spp. had an increased prevalence after 4 days of storage, their relative share was lower than in pure carrot juice; this was especially noticeable in the C+CBD variant. Interestingly, in the same variant, the *Yersinia* spp. was significantly higher, at over 40% of relative abundance in the juice microbiome.

For the evaluation of α-biodiversity, three determinants based on the OTU number were determined: Chao-1 (richness estimation), Shannon (diversity index), and Simpson (evenness index). Their values are presented in Table 1. While it is clear that the microbiome’s richness increased with the storage time, the addition of vanilla and mint extracts further increased it. The metapopulation diversity increased alongside richness across all carrot juice variants; however the pure carrot juice was the most diverse of the samples. Interestingly, the results do not indicate any significant changes occurring in the microbiome’s evenness, as the Simpson index remained relatively stable across all samples during the 4 days of storage.

**Figure 3:**
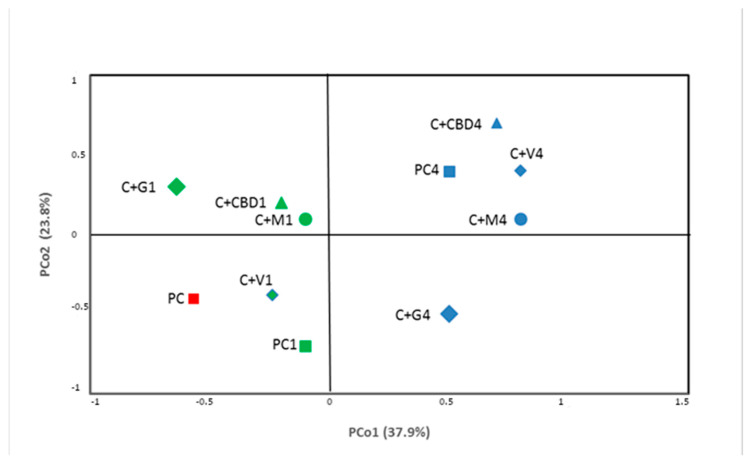
Principal coordinates analysis (PCoA) of carrot juice variants microbiome.

Figure 3 presents the β-biodiversity of the carrot juice variants microbiome. The β-biodiversity describes the similarities between metapopulational profiles with the use of principal component analysis (PCoA). Roughly four groups with a high level of similarity can be discerned from this analysis. The main differentiative factor appeared to be the time of storage, as samples from after the 1st day and 4th day of storage were significantly different. Interestingly, the C+V variant, after the first 24 h of storage, was relatively similar to the pure carrot juice, which also underwent relatively small changes during that time. Secondly, the C+G variant showed a small similarity with the rest of the carrot juice variants sampled after 4 days of storage.

#### 2.1.2. Microbiological Analyses

The total mesophilic and psychrophilic bacterial counts of carrot juice are shown in Table 2 and Table 3, respectively. The total mesophilic bacteria count for pure carrot juice at the time of preparation was 4.1 log CFU/mL. The total psychrophilic bacteria count of all juice variants at the time of preparation was similar to the total mesophilic count. In the case of juice variants C+M and C+CBD, the total mesophilic count on the day of preparation was similar to pure carrot juice, while, for variants C+V and C+G, the addition of, respectively, vanilla extract and grapefruit extract decreased the microbial count by one logarithmic cycle. For all juice variants, the total mesophilic bacteria count had not changed after the 2 standard days of storage; however, it increased after 4 days of storage.

As can be seen in Table 4, the addition of vanilla extract and grapefruit extract has caused a decrease in the Enterobacteriaceae count, from 4.1 log CFU/mL to 3.4 and 3.14 log CFU/mL, respectively. After two days of storage, the Enterobacteriaceae count did not differ significantly from the initial count, with the exception of the C+M variant. An increase was noted in all variants after the first 24 h of storage, and, interestingly, also after 4 days of storage, which was significant only in C+M and C+CBD.

The total coliform count is presented in Table 5. At the time of preparation, the coliform count for fresh, pure carrot juice was 3.43 log CFU/mL. Analogically to Enterobacteriaceae count, the addition of vanilla extract and grapefruit extract to carrot juice caused a decrease in coliform count. Interestingly, the addition of mint extract and CBD oil caused an increase of one logarithmic cycle in the coliform count in carrot juice. The total coliform count decreased significantly after the second day of storage, and it is interesting to notice that, after 4 days of storage, in all carrot juice variants containing potentially antimicrobial additives, the increase in the coliform count was noticeably smaller than in pure carrot juice.

The changes in total *Pseudomonas* count are presented in Table 6. The addition of mint extract and CBD oil to carrot juice caused an increase in *Pseudomonas* count by one logarithmic cycle compared to pure carrot juice on day “0”. At the time of preparation, the total *Pseudomonas* count in pure carrot juice was 3.3 log CFU/mL. Unlike the total Enterobacteriaceae or coliforms counts, the addition of vanilla extract and grapefruit extract did not cause a significant decrease in *Pseudomonas* count. Changes in *Pseudomonas* count were observed after the second day of storage, but a strong increase was only noted in pure carrot juice. However, after 4 days of storage, the *Pseudomonas* count had increased across all variants of carrot juice.

As presented in Table 7, the addition of mint extract, vanilla extract, and CBD oil to carrot juice caused a significant decrease in the total yeast and mold count, as none were identified at the time of preparation in those carrot juice variants. The addition of grapefruit extract did not significantly affect the total yeast and mold count in carrot juice, which in fresh, pure carrot juice was 1.71 log CFU/mL. The total yeast and mold count increased during the 48 h of storage in all variants of carrot juice. Interestingly, yeasts and molds were not detected in either variant after 4 days of storage.

### 2.2. Physicochemical Analyses

#### pH Analysis

The changes in the carrot juice’s pH during storage are presented in Table 8. The pH of fresh, pure carrot juice (PC) was 6.55 at the time of preparation. The pH of carrot juice with added potential antimicrobials significantly decreased after mixing the components. It has to be noted that, despite the decrease in the overall pH of all carrot juice variants, the pH remained within the slightly acidic–neutral range. The carrot juice variant with the lowest pH was carrot juice with mint extract (C+M) at 6.30. The pH of all carrot juice variants researched in this work remained relatively stable during the 48 h of storage; after 4 days of storage the pH decreased in all variants; notably, in the C+M variant, the decrease was not statistically significant.

## 3. Discussion

### 3.1. Microbiome Analyses

Minimally processed food of plant origin is easily contaminated with microorganisms during all of the stages of its production: pre- and post-harvest, transport, processing, etc. There are many possible sources of contamination: soil, fertilizers, or water. During processing, the possibility of cross-contamination also arises. Minimally processed foods, and, especially, foods of plant origin that are usually consumed fresh, have a high risk of contamination with pathogens such as *Salmonella* sp., *Escherichia* spp., *Bacillus* spp., or *Listeria monocytogenes.* According to EFSA (European Food Safety Authority), the risk of foodborne diseases has been growing in recent years, which indicates the need not only for constant quality control but also for rapid quality control [31]. According to European Commission Regulation No 2073/2005 [32], there are two criteria applicable to fresh, unpasteurized vegetable juice: *Salmonella* sp. cannot be found in 25 g of product, and the maximum limit of *Escherichia coli* found in the product is 3.1 log CFU/g. No bacteria belonging to the genera *Salmonella* sp. or *Escherichia* spp. were found in the analyzed carrot juice variants. However, some of the bacteria identified both in fresh, pure carrot juice, as well as in fresh carrot juice with additives, are potentially pathogenic or pathogenic to humans. Among them, *Achromobacter* spp., *Enterobacter* spp., *Pseudomonas* spp., *Sphingomonas* spp., or *Yersinia* spp. can be mentioned. However, fresh produce or minimally processed fresh produce are often considered as spoiled and not viable for consumption when their microbial load exceeds 7.0 log CFU/g [33]. The standard methods of microbial analysis performed in this research show that, during the 4 days of storage, none of the analyzed carrot juice variants exceeded this limit. While next-generation sequencing has allowed for the qualitative analysis of carrot juice’s microbiome and has proven that the microbiological quality of unpasteurized carrot juice may be considered questionable due to pathogens found in it, the standard method of analysis has shown that, technically, the analyzed juice was not spoiled after 4 days of storage.

The analysis of carrot juice samples has shown that the first 24 h of storage affected the metapopulation of carrot juice by increasing the number of dominant bacterial families present in it. The juice was also affected by the addition of antimicrobial agents. However, while variants of carrot juice with additives differ in their microbiome composition from pure carrot juice, the increasing trend in species richness and diversity is clear across all samples. The time of storage appeared to be the most differentiative factor between the analyzed samples. Antimicrobials have generally increased the carrot juice microbiome diversity further, although they have not affected the microbiome’s evenness. After 4 days of storage, the Enterobacteriaceae and Pseudomonadaceae families, and, in the case of the C+G variant, also the Rivulariaceae family, were dominating the carrot juice microbiome in all samples. It is important to note that many of the genera identified in this research are pathogenic or potentially pathogenic. Of the 33 most prevalent genera, at least 14 are pathogenic or opportunistically pathogenic; among them, such microorganisms as *Achromobacter*, *Sphingomonas*, *Ochrobactrum*, *Pseudomonas*, *Stenotrophomonas*, *Klebsiella*, *Enterobacter*, *Mycoplasma*, *Serratia* and *Yersinia* spp. could be mentioned. The microbiome of carrot juice consisted of bacteria usually associated with soil and groundwater, since those are common in the rhizosphere of carrot roots and vegetables in general, as has been shown by [34]. Ekici et al. [35] have observed a clear dominance of lactic acid bacteria in researched fermented carrot juice; however, these bacteria were part of the starter. Interestingly, they also noticed a relatively high prevalence of the Enterobacteriaceae family, and the Enterobacter genus in particular, in the carrot juice, even after several days of fermentation. However, Van Beeck et al. [36] have noticed that, while the Enterobacteriaceae family (mostly *Enterobacter* and *Yersinia* spp.) dominated the fresh carrot juice microbiome, they were outcompeted by lactic acid bacteria as the fermentation of their juice progressed. The differences between these experiments may lie in the initial levels of microbial contamination of carrot juice, and also in the addition of starter culture—for this research, the fact that fermentation may not completely inhibit the growth of potential pathogens in carrot juice is important. Hussain et al. [37] researched the metapopulation of fruit juices and found *Sphingomonas* to be one of the most dominant genera. Interestingly, among 51 genera identified by them, around 11 were pathogenic, and, among them, *Stenotrophomonas, Klebsiella*, and *Ochrobactrum* bacteria were also present.

The total mesophilic bacteria count for pure carrot juice observed at the time of preparation puts it in the usual range of TMB count for fresh carrot juice [38,39,40]. Ben-Fadhel et al. [41] have noted a decrease of almost two logarithmic cycles in the TMB count in fresh carrots after treatment with antimicrobial formulation (mixture of EOs), which is similar to the result obtained in this work. The total mesophilic bacteria count in all juice variants increased noticeably after 4 days of storage, and microbial spoilage appeared to be even across all variants. Interestingly, Gottardi et al. [38] also did not observe a significant change in the microbial quality of fresh carrot juice during 48 h of storage—in fact, the TMB count of pure carrot juice observed after 2 days of storage was lower than the initial one; however, the TMB of their juice had exceeded 7.0 log CFU/mL after 9 days of storage, which generally indicates the state of microbial spoilage in fresh produce or its direct derivatives. Interestingly, Leneveu-Jenvrin et al. [42] observed that commercial carrot juice presented much better microbial quality by TPB count than TMB count, as, even after three days of storage, it did not exceed 5.0 log CFU/mL.

Leneveu-Jenvrin et al. [42] observed the total Enterobacteriaceae count at over 5 log CFU/mL for freshly prepared pure carrot juice, and over 5.5 log CFU/mL for commercial fresh carrot juice. They also noticed that, during 3 days of storage, the total Enterobacteriaceae count in lab-made carrot juice was stable, while, in commercial carrot juice, it increased by almost one logarithmic cycle. In this experiment, the Enterobacteriaceae count increased significantly in all carrot juice variants during the first 24 h of storage. Interestingly, the increase in the Enterobacteriaceae count observed after 4 days of storage was less significant, despite the signs of microbial spoilage of the juice. Leneveu-Jenvrin et al. [42] observed that the Enterobacteriaceae count increased significantly between day 3 and day 7 of storage; however, there was little to no change between day 7 and day 10 of storage.

The coliform count for fresh, pure carrot juice at the time of preparation was almost identical to the results obtained by Gottardi et al. [38] and almost two logarithmic cycles higher than the results obtained by Rodriguez et al. [40]. Gottardi et al. [38] also noticed that the total coliform count in analyzed pure carrot juice was stable during 48 h of storage. The total coliform count decreased significantly after the second day of storage. However, it is interesting to note that the coliform count increased again after 4 days of storage, yet, in all carrot juice variants containing potentially antimicrobial additives, the increasing trend was lesser than in pure carrot juice.

Yeasts and molds were not detected at the time of preparation in carrot juice variants with added potential antimicrobial agents, with the exception of the C+C variant. Bevilacqua et al. [43], in their study on the effect of essential oil addition to fresh juices, have noted that yeasts and molds are usually the most prone to inactivation by antimicrobial agents, while Gram-negative bacteria (such as most of the Enterobacteriaceae group) appear to be much more resistant, possibly due to their cell wall and membrane structure. Rodriguez et al. [40] observed a total yeast and mold count in their fresh, pure carrot juice that was nearly identical to the count observed in this experiment. Adversely, Leneveu-Jenvrin et al. [42], during their analysis of lab-made fresh carrot juice and commercial fresh carrot juice, noted that both had significantly higher total yeast and mold counts—around 4.7 and 5.7 logs CFU/mL, respectively, and, after 10 days of storage, that count had further increased. In this experiment, the total yeast and mold count increased in all carrot juice variants during the first 2 days of storage, but, after 4 days of storage, yeasts and molds were again absent from the juice.

Essential oils (EOs) are considered to possess a strong and wide-spectrum antimicrobial activity, and this research has proven they have an inhibitory effect against a wide range of microorganisms present in unpasteurized carrot juice. However, the concentration needed for their antimicrobial activity to take effect in the final product is often too high, which results in lowering the product’s sensorial quality and, therefore, its acceptability to consumers. However, there is a high potential for obtaining effective techniques by combining EOs with non-thermal technologies such as ultrasound, irradiation, or ozone. Such combinations were proven to have a synergistic antimicrobial effect on many food products, including juice [44]. The second possibility lies in combining various several essential oils, each at a relatively low concentration, for a stronger antimicrobial effect. This has been proven to be an effective method against *E. coli* O157:H7 on fresh-cut lettuce [45]. Additionally, György et al. [46] have also proven that some combinations of EOs, such as mint essential oil combined with oregano or thyme essential oil, are effective against microorganisms isolated from fresh produce and are good candidates for practical application. Menthol, one of the antimicrobial compounds found in peppermint EO, was also proven to be effective against, e.g., Listeria monocytogenes and Bacillus cereus in a mixture of four EOs [47]. It has to be mentioned that a combination of essential oils used at lower concentrations has been proven to be more acceptable to consumers [48].

### 3.2. Physicochemical Analyses

#### pH Analysis

Carrot juice is considered an outlier among other commercially available juices due to its naturally high pH. The changes in the juice pH are connected with the degradation, especially microbial degradation, occurring in the product despite the addition of potential antimicrobial agents. The pH of fresh, pure carrot juice on the day of preparation was 6.55, which is higher than the pH of commercial carrot juice (6.2) researched by Schultz et al. [49] in their work, the carrot juice (6.3) prepared by Amanyunose et al. [50] in their work, and the commercial carrot juice (6.4) researched by Pokhrel et al. [1] in their work. However, Rodriguez et al. [40] obtained fresh, pure carrot juice with a pH value of 6.52, which is similar to the pH of carrot juice obtained in this work. The addition of potential antimicrobials significantly decreased the pH of all carrot juice variants, most likely due to their more acidic pH values. Pokhrel et al. [1] and Leahu et al. [51] both observed a decrease in the pH of carrot juice blended with other juices—orange and apple juice, respectively. The pH of all carrot juice variants, including the reference juice analyzed in this work, remained relatively stable between day 1 and day 2 of storage, and, after 4 days, the pH decreased in all variants sans C+M variant. The pH of pure carrot juice and carrot juice with *Aframomum danielli* extract decreased during storage at 4 °C in the research conducted by Amanyunose et al. [50]. Similarly, in the research of Pokhrel et al. [1], the pH of the carrot and orange juice blend decreased during five days of storage. And while Gottardi et al. [38] observed a trend of slightly increasing pH value during the first 6 days of storing pure, non-pasteurized carrot juice at 4 °C, after 12 days, the pH had decreased significantly.

## 4. Materials and Methods

### 4.1. Sample Procurement

The analyses were carried out using carrots obtained in the local market. The carrots (a variety undisclosed by the producer) were cultivated in Colonia Monte Algaida, Sanlúcar de Barrameda, Spain by Agricola Del Sur S XXI SL with organic farming practices. The carrots were harvested in June 2022. Antimicrobial agents used as additives to juice were: food-grade Madagascar bourbon vanilla extract (Nielsen-Massey, Waukegan, IL, USA), food-grade peppermint extract (Nielsen-Massey), HempExtract 300 mg CBD (Hempbroker Polska, Wrocław, Poland), and concentrated grapefruit extract.

### 4.2. Juice Preparation

Carrots were pre-washed with tap water, then hand peeled, washed again, and blotted dry. The carrot juice was extracted from prepared vegetables using a commercial juicer (BRAND). Obtained juice was stored for 30 days at 6 °C in glass bottles of 500 mL capacity; each bottle contained 300 mL of juice. Samples were taken on days 0, 1, 2, and 4.

The prepared carrot juice was divided into one reference variant and four variants of juice with additives: pure carrot juice, carrot juice with bourbon vanilla extract added to final 5% (*v*/*v*) concentration (C+V variant), carrot juice with peppermint extract added to final 5% (*v*/*v*) concentration (C+M variant), carrot juice with CBD oil added to final 0.5% (*v*/*v*) concentration (C+CBD variant), and carrot juice with concentrated grapefruit extract added to final 5% (*v*/*v*) concentration (C+G variant). The potential antimicrobials were added in their original form, that is, in the form provided by their respective manufacturers without further dilutions or other changes. All of the additives were filtered with the use of Millex sterile syringe filters with 0.10 µm pore size (Merck Millipore, Burlington, VT, USA) and tested for microbial contamination in accordance with ISO standards. All of the additives tested negative for total mesophilic and psychrophilic microorganism count and total Enterobacteriaceae count.

### 4.3. Physicochemical Analyses

#### pH Analysis

The pH of the carrots was measured using a CP-401 pH meter (Elmetron, Zabrze, Poland). The pH meter electrode was placed in 20 mL of analyzed carrot juice. Measurements were conducted in triplicate for each sample.

### 4.4. Microbiome Analyses

#### 4.4.1. Next-Generation Sequencing Analysis

For this analysis, 11 samples were chosen: freshly prepared pure carrot juice (PC) as a reference sample from day “0”; pure carrot juice (PC1) sample and carrot juice variants with added potential antimicrobial agents (C+V1, C+M1, C+CBD1, C+G1) samples from day “1”; and pure carrot juice (PC4) sample and carrot juice variants with added potential antimicrobial agents (C+V4, C+M4, C+CBD4, C+G4) samples from day “4”.

##### DNA Isolation

The DNA isolation from juice samples (3 mL of each sample) was performed using the Genomic Mini AX Bacteria Spin Kit (A&A Biotechnology, Gdynia, Poland). The procedure was carried out according to the instructions provided and recommended by the manufacturer. Isolate concentration was assessed by a fluorimetry method on the Qubit 3.0 device (ThermoFisher Scientific, Waltham, MA, USA) with the use of the dsDNA HS Assay Kit (ThermoFisher Scientific). For each of the samples, three DNA extractions were performed and later combined after a positive quantification.

##### PCR Amplification

The V4 region of the bacterial 16s rRNA was amplified with the use of 515F-806R primers described by Caporaso et al. [52]. The PCR reaction mixture contained: 12.5 µL PCR Master Mix containing Taq Polymerase (ThermoFisher Scientific), 2.5 µL nuclease-free water (ThermoFisher Scientific), 5 µL of each primer, and 5 µL of genomic DNA. PCR amplification was performed using the following conditions: initial denaturation at 95 °C for 3 min; 35 cycles of denaturation at 95 °C for 1 min; annealing of the primers at 52 °C for 30 s and elongation at 72 °C for 1 min; then final extension at 72 °C for 10 min. The PCR products were purified using a Clean-Up column (A&A Biotechnology) according to the manufacturer’s protocol.

##### Sequencing

Amplicon sequencing was performed with the use of the MiSeq platform (Illumina, San Diego, CA, USA). Amplicons were constructed with the use of the NEBNext^®^ DNA Library Prep Master Mix Set for Illumina (New England Biolabs, Ipswich, MA, USA) according to the manufacturer’s protocol. Libraries were normalized to equimolar concentrations and quantified with a fluorimetry technique on Qubit 3.0 Fluorometer (Invitrogen, Waltham, MA, USA) using the dsDNA HS Assay Kit (Life Technologies, Carlsbad, CA, USA). The libraries were denatured in the presence of 0,2 N NaOH and diluted with HT1 buffer (Illumina) to a final concentration of 8 pM. Libraries were sequenced on a MiSeq platform (Illumina) using the MiSeq Reagent Kits v2 (Illumina) with the same primers used as in the previous PCR reaction.

##### Bioinformatic Analysis and Visualization

Bioinformatic analysis of the raw sequencing data was performed using CLC Genomics Workbench 20.0 with CLC Microbial Genomics Module 20.1.1 software (Qiagen, Hilden, Germany) in a procedure used by Hornik et al. [53]. The readings were compared with the SILVA v119 database on the basis of 97% sequence similarity of the operational taxonomic units (OTU) [54]. α-biodiversity indicators such as OTU number, Chao1 index, Shannon function, and Simpson index were determined according to the procedure described by [55].

#### 4.4.2. Microbiological Analysis

Determination of microbiological quality was carried out using a standard method in accordance with ISO standards as described by Ratajczak et al. [56]. The measurements were conducted for the total number of mesophilic and psychrophilic microorganisms (medium used was standard agar with glucose and nutrient broth), the number of Enterobacteriaceae (medium used was VRBG agar, BTL), the number of coliforms (medium used was MacConkey agar, BTL), the number of *Pseudomonas* (medium used was CFC agar, BTL, with *Pseudomonas* CFC Supplement, BTL, added), and the number of yeasts and molds (medium used was DRBC agar, GRASO Biotech). In order to carry the procedure decimal, dilutions of analyzed carrot juice were prepared using a sterile saline solution. All inoculations were carried out using the pour plate procedure.

## 5. Conclusions

The addition of potential antimicrobials improved the overall microbial quality of unpasteurized carrot juice during the 48 h of cold storage. Fresh, unpasteurized carrot juice suffers from high microbial contamination and, thus, fast spoilage processes that render it unacceptable for consumption after 24 h. However, the addition of antimicrobials generally improved the initial microbial quality and also slowed the growth of microorganisms present in carrots, thus resulting in the prolongation of the fresh juice shelf-life by up to 48 h. It is interesting to note that each of the antimicrobial agents had a different effect on carrot juice’s microflora—peppermint extract appeared to be especially effective against Enterobacteriaceae bacteria and yeasts and molds, while vanilla extract and grapefruit extract were more effective against coliforms and *Pseudomonas* bacteria. Importantly, other research has shown that it is possible to combine different essential oils for a stronger antimicrobial effect. Similarly, it is possible to combine essential oils with non-thermal sanitizing techniques. This means that combining the antimicrobial agents used in this study may prove to be a promising way of enhancing the quality of unpasteurized carrot juice. However, next-generation sequencing analysis has proven that, while antimicrobials have affected the microbiome of pure carrot juice, they were not capable of enhancing the microbiome composition or preventing potential pathogenic bacteria from occurring in juice samples. The addition of potential antimicrobials did not significantly affect the pH of carrot juice during storage, but, before this method can be considered for practical application, a more in-depth analysis of the product’s physicochemical properties, including a sensorial analysis, should be conducted.

In conclusion, the addition of antimicrobial agents appears to be a promising technique for improving the quality, especially microbial quality, of fresh carrot juice. The conducted research opens the way for further studies on the efficiency of the antimicrobials for use in carrot juice and different products of plant origin, and it may become crucial for control over food safety.

## Figures and Tables

**Table 1:** Values of α-biodiversity indicators of the carrot juice variants microbiome.

Index	PC	PC1	C+V1	C+M1	C+CBD1	C+G1	PC4	C+V4	C+M4	C+CBD4	C+G4
OTU	126.00	148.00	169.00	157.00	187.00	134.00	269.00	287.00	291.00	246.00	214.00
Chao-1	134.00	165.00	184.00	169.00	201.00	154.00	288.00	302.00	316.00	266.00	238.00
Shannon	2.87	2.95	3.14	3.21	3.08	2.98	4.89	4.71	4.51	4.77	4.19
Simpson	0.84	0.86	0.83	0.84	0.85	0.83	0.84	0.81	0.83	0.84	0.82

**Table 2:** Changes in the total mesophilic bacteria count in carrot juice during storage.

Time	PC	C+V	C+M	C+G	C+CBD
[Days]	[log CFU/mL]				
0	4.10 ± 0.01 a	3.90 ± 0.00 b	4.30 ± 0.02 c	3.25 ± 0.00 d	4.30 ± 0.01 c
1	4.14 ± 0.01 a	3.80 ± 0.01 b	4.10 ± 0.02 a	3.13 ± 0.00 c	4.10 ± 0.01 a
2	4.68 ± 0.02 a	3.90 ± 0.01 b	3.35 ± 0.01 c	3.61 ± 0.01 d	3.47 ± 0.01 e
4	5.60 ± 0.01 a	4.74 ± 0.01 b	4.65 ± 0.01 c	4.90 ± 0.01 d	4.22 ± 0.02 e

In each row, means with different letters are significantly different (*p* = 0.05).

**Table 3:** Changes in the total psychrophilic bacteria count in carrot juice during storage.

Time	PC	C+V	C+M	C+G	C+CBD
[Days]	[log CFU/mL]				
0	3.90 ± 0.01 a	3.30 ± 0.00 b	4.20 ± 0.01 c	3.11 ± 0.01 d	4.20 ± 0.00 c
1	3.97 ± 0.03 a	4.43 ± 0.04 b	4.10 ± 0.00 c	3.15 ± 0.00 d	4.50 ± 0.00 e
2	5.11 ± 0.01 a	4.44 ± 0.03 b	3.41 ± 0.01 c	4.99 ± 0.01 d	4.60 ± 0.01 e
4	5.50 ± 0.00 a	5.90 ± 0.01 b	4.27 ± 0.01 c	5.80 ± 0.00 d	6.61 ± 0.01 e

In each row, means with different letters are significantly different (*p* = 0.05).

**Table 4:** Changes in the total Enterobacteriaceae count in carrot juice during storage.

Time	PC	C+V	C+M	C+G	C+CBD
[Days]	[log CFU/mL]				
0	4.10 ± 0.00 a	3.40 ± 0.00 b	4.20 ± 0.00 c	3.14 ± 0.01 d	4.10 ± 0.00 a
1	6.50 ± 0.01 a	3.42 ± 0.02 b	4.70 ± 0.00 c	4.16 ± 0.01 d	6.21 ± 0.01 e
2	5.10 ± 0.05 a	3.82 ± 0.02 b	3.19 ± 0.03 c	3.80 ± 0.00 b	3.18 ± 0.02 c
4	6.11 ± 0.03 a	4.48 ± 0.02 b	4.15 ± 0.02 c	4.90 ± 0.01 d	5.10 ± 0.01 e

In each row, means with different letters are significantly different (*p* = 0.05).

**Table 5:** Changes in the total coliform count in carrot juice during storage.

Time	PC	C+V	C+M	C+G	C+CBD
[Days]	[log CFU/mL]				
0	3.43 ± 0.03 a	2.58 ± 0.01 b	4.10 ± 0.01 c	2.27 ± 0.01 d	4.20 ± 0.00 e
1	2.19 ± 0.01 a	2.73 ± 0.02 b	4.10 ± 0.00 c	3.40 ± 0.00 d	5.11 ± 0.01 e
2	2.71 ± 0.19 a	1.80 ± 0.14 b	2.20 ± 0.32 c	2.34 ± 0.21 a	3.34 ± 0.13 c
4	5.23 ± 0.04 a	3.52 ± 0.06 b	3.87 ± 0.18 c	3.30 ± 0.05 d	4.10 ± 0.04 e

In each row, means with different letters are significantly different (*p* = 0.05).

**Table 6:** Changes in the total *Pseudomonas* count in carrot juice during storage.

Time	PC	C+V	C+M	C+G	C+CBD
[Days]	[log CFU/mL]				
0	3.30 ± 0.00 a	3.30 ± 0.00 a	4.20 ± 0.00 b	3.11 ± 0.03 c	4.10 ± 0.00 d
1	3.40 ± 0.02 a	3.39 ± 0.01 a	4.10 ± 0.02 b	3.22 ± 0.02 c	4.20 ± 0.00 d
2	4.48 ± 0.02 a	3.62 ± 0.01 b	3.37 ± 0.01 c	3.31 ± 0.03 d	3.51 ± 0.01 e
4	4.84 ± 0.03 a	4.11 ± 0.01 b	3.52 ± 0.01 c	3.86 ± 0.01 d	4.12 ± 0.01 b

In each row, means with different letters are significantly different (*p* = 0.05).

**Table 7:** Changes in the total yeast and mold count in carrot juice during storage.

Time	PC	C+V	C+M	C+G	C+CBD
[Days]	[log CFU/mL]				
0	1.71 ± 0.01 a	0 b	0 b	1.40 ± 0.01 c	0 b
1	2.40 ± 0.00 a	1.10 ± 0.00 b	0 c	2.53 ± 0.01 d	0 c
2	2.71 ± 0.01 a	1.80 ± 0.00 b	2.20 ± 0.00 c	3.10 ± 0.00 d	3.18 ± 0.00 e
4	0 a	0 a	0 a	0 a	0 a

In each row, means with different letters are significantly different (*p* = 0.05).

**Table 8:** Changes of pH value of carrot juice variants during storage.

Time	PC	C+V	C+M	C+G	C+CBD
[Days]					
0	6.55 ± 0.01 a	6.38 ± 0.01 b	6.31 ± 0.01 c	6.34 ± 0.01 d	6.30 ± 0.01 c
1	6.74 ± 0.01 a	6.55 ± 0.01 b	6.39 ± 0.02 c	6.42 ± 0.01 c	6.46 ± 0.02 d
2	6.75 ± 0.01 a	6.55 ± 0.02 b	6.42 ± 0.01c	6.59 ± 0.01 d	6.47 ± 0.02 e
4	6.65 ± 0.01 a	6.52 ± 0.02 b	6.41 ± 0.01c	6.44 ± 0.01 d	6.32 ± 0.02 e

In each row, means with different letters are significantly different (*p* = 0.05).

## Data Availability

Data sharing is not available for this article.

## Sample Availability

Samples of the analyzed carrot juices are not available from the authors.
